# Biomass and Cordycepin Production by the Medicinal Mushroom *Cordyceps militaris*—A Review of Various Aspects and Recent Trends towards the Exploitation of a Valuable Fungus

**DOI:** 10.3390/jof7110986

**Published:** 2021-11-19

**Authors:** Dimitrios Kontogiannatos, Georgios Koutrotsios, Savvina Xekalaki, Georgios I. Zervakis

**Affiliations:** Laboratory of General and Agricultural Microbiology, Agricultural University of Athens, Iera Odos 75, 11855 Athens, Greece; georgioskoutrotsios@gmail.com (G.K.); savinax.96@gmail.com (S.X.)

**Keywords:** *Cordyceps militaris*, *Ophiocordyceps sinensis*, functional food, nutraceuticals, medicinal mushroom, cordycepin

## Abstract

*Cordyceps militaris* is an entomopathogenic ascomycete with similar pharmacological importance to that of the wild caterpillar fungus *Ophiocordyceps sinensis*. *C. militaris* has attracted significant research and commercial interest due to its content in bioactive compounds beneficial to human health and the relative ease of cultivation under laboratory conditions. However, room for improvement exists in the commercial-scale cultivation of *C. militaris* and concerns issues principally related to appropriate strain selection, genetic degeneration of cultures, and substrate optimization. In particular, culture degeneration—usually expressed by abnormal fruit body formation and reduced sporulation—results in important economic losses and is holding back investors and potential growers (mainly in Western countries) from further developing this highly promising sector. In the present review, the main factors that influence the generation of biomass and metabolites (with emphasis on cordycepin biosynthesis) by *C. militaris* are presented and evaluated in conjunction with the use of a wide range of supplements or additives towards the enhancement of fungal productivity in large-scale cultivation processes. Moreover, physiological and genetic factors that increase or reduce the manifestation of strain degeneration in *C. militaris* are outlined. Finally, methodologies for developing protocols to be used in *C. militaris* functional biology studies are discussed.

## 1. Introduction

*Cordyceps* is a large and diverse genus of the family Cordycipitaceae (Hypocreales, Ascomycota) comprising 627 species according to MycoBank (https://www.mycobank.org; 12 November 2021). They are parasitic fungi, mostly endoparasitoids of insects and other arthropods, while some of them are parasitic on other fungi [1]. *Cordyceps* was named after the Greek word “kordyle” meaning “club”, and the Latin stem “-*ceps*” meaning “head” [2]. Most species grow on larvae, pupae, or adults of insects of the orders Arachnida, Coleoptera, Lepidoptera, Hymenoptera, Hemiptera, Orthoptera, Diptera, and Isoptera [3]. *Cordyceps militaris* (L.) Fr. is a common endoparasitoid of insects, usually growing on pupae and less commonly found on larvae, and is widely distributed in North and South America, Europe, and Asia. Fruit bodies (ascomata) are variable in form and especially in size; this variability is associated with the type of food source(s) available for fungal growth, while it is also affected by the size of the host and the number of stromata formed [3].

Fungi of the genus *Cordyceps* are among the most important “traditional medicines” since they contain bioactive compounds of high pharmacological value. Fruit bodies are traditionally collected in the wild, while some species are cultivated for the production of mycelium and ascomata [2]. *C. militaris* is the most studied member of the genus popularly referred to as “Bei Chong Cao” in China. It ranks second (after *Ophiocordyceps sinensis*) among the most commercialized *Cordyceps* species in East Asia, where it is used as folk tonic medicine and is considered as a cheaper substitute for *O. sinensis* [2,4]. Moreover, *C. militaris*-related products are popular nutraceuticals consumed either as culinary mushrooms or sold in several other forms, e.g., extracts, fermented powder, and tinctures. As regards the medicinal properties that have been attributed to *C. militaris*, they include hypoglycemic, hypolipidemic, anti-inflammatory, antitumor, antibacterial, antifungal, antiviral, antimalarial, prosexual, neuroprotective, antioxidant, and immuno-protective activities [2,5,6]. Many bioactive molecules of nutraceutical interest, such as cordycepin, ergosterol, trehalose, mannitol and several polysaccharides, nucleosides, and amino acids have been detected in (or isolated from) *C. militaris* [2,5,7]. Among them, cordycepin is considered as the most valuable and economically important compound produced by this fungus. Particularly noteworthy is the recent discovery of a ProTide modified form of cordycepin named NUC-7738, which presents up to 40 times greater potency for killing cancer cells than the original compound [8]. Such findings will most certainly have to be accompanied/supported by the necessary technological advancements in large-scale *C. militaris* biomass production to meet increased demand in cordycepin.

*C. militaris* mycelia and ascomata can be generated ex situ with or without the addition of alive and/or dead insect tissues. A wide range of methodologies to produce fruit bodies and mycelia of *C. militaris* are reported in pertinent literature including: (a) solid-state fermentation [9,10,11,12], (b) submerged static fermentation [11,13], (c) repeated batch or one-step non-static fermentation in liquid media [14,15,16], and (d) cultivation following larval or pupal infection [17,18]. However, commercial exploitation of *C. militaris* is still in need of improvement in several aspects related to fruit body production, metabolite biosynthesis, and fungal pathogenicity to target insects. Strain degeneration is the most essential since it significantly influences *C. militaris* productivity, causing considerable economic losses at the industrial level [19,20]. Several factors are involved in *C. militaris* degeneration that are either intrinsic/genetic or cultivation-related [20], as presented in the next section.

Furthermore, in order to optimize *C. militaris* productivity, many parameters have already been examined through numerous pertinent studies that will be suitably outlined and discussed in the present review. Most of them focused on developing suitable methodologies and interventions to avoid (or minimize) culture degeneration [21,22,23,24,25,26,27,28,29,30,31], on improving ascomata and mycelia production [12,15,32,33,34,35,36], and/or on enhancing cordycepin output by adopting various approaches and the use of both solid and liquid substrates [12,15,23,32,37,38,39,40,41,42,43,44,45,46,47]. In addition, the application of genetic engineering breakthrough technologies has resulted in a considerable improvement in *C. militaris* biomass content in various metabolites, including cordycepin [37,48,49,50,51].

In this review, we present data related to key factors influencing biomass and metabolite production in *C. militaris* by placing emphasis on culture degeneration, substrate composition, and the use of supplements. Moreover, we discuss genetic and cultivation aspects that affect cordycepin biosynthesis and ascomata formation in *C. militaris* as well as functional genomics and genetic engineering processes that could substantially enhance the exploitation of this fungus and the generation of value-added products.

## 2. The Effect of Culture Degeneration on the Yield and Quality of Fruit Bodies Produced by *C. militaris*

It is widely known that fungi lose virulence and that their morphology is altered when they are successively subcultured on artificial media. Various terms have been used to describe this phenomenon, including phenotypic degeneration, phenotypic instability or deterioration, dual phenomenon, saltation, and attenuation [52,53,54,55,56]. Morphological changes include modifications in color and growth form as well as reduced sporulation [53]. As regards *C. militaris* in particular, degenerated cultures demonstrate slower mycelial growth, a lighter color of hyphae (probably due to decrease in pigment content), decrease in the number of primordia formed, reduced (or no) ability to produce fruit bodies, longer growth cycle, abnormal fruit body formation, decrease in conidia production, decrease in secondary metabolites content, lower dehydrogenase activity, and reduced cellulase and amylase activities, as well as lower extracellular and higher intracellular polysaccharide content [20,21,22,24,57,58]. 

He et al. [25] evaluated the effect of mineral elements on colony types of *C. militaris* and concluded that K^+^, Ca^2+^, and Zn^2+^ (at concentrations of 1.0 g L^−1^, 0.02 g L^−1^, and 250–375 μg L^−1^, respectively) delayed, whereas Mn^2+^ and Mg^2+^ (at trace concentrations) promoted the degeneration of *C. militaris* (Table 1), In addition, degeneration of *C. militaris* was affected by oxidative stress [20,22], and it was promoted by continuous subculturing, which became particularly evident in the fourth and the fifth generations [20,58]. Furthermore, homokaryosis seems to promote degeneration of *C. militaris* [1,20,26]; however, degenerated strains could be rejuvenated by cross-mating of their single ascospore isolates [20,24] (Table 1).

In general, few data exist about the effect of the type of substrate (and the nutrients it contains) on degeneration of *C. militaris*. However, it is expected that this entomopathogenic fungus could benefit by being grown on substrates containing insect cells or tissues since such media emulate better the natural host’s environment. For example, when tussah pupae were inoculated by degenerated cultures of *C. militaris*, the rate of mycelial growth and the yield of fruit bodies were improved [59]. Although such results indicate that the use of insect tissues could enhance biomass production by *C. militaris*, further studies are needed to better understand their effect on the cultivation process.

A rather limited amount of information is available about molecular mechanisms affecting *C. militaris* culture degeneration. Apparently mating-type (MAT) loci allele segregation—through continuous culture—is closely related to the manifestation of this phenomenon [37,58,60,61,62,63,64]. In addition, gene mutation [31] and DNA methylation [27,30] also seem to be involved by inducing strain degeneration. Yin et al. [58] performed a transcriptome-wide analysis during *C. militaris* subculturing and found that genes implicated in detoxification and stress response mechanisms (such as those involving the production of streptothricinacetyltransferase, gamma-glutamyltranspeptidase, MFS multidrug transporter, glutathione S-transferase, alcohol dehydrogenase, and the 30 kDa heat shock protein) were up-regulated during strain degeneration. Similarly, expression of genes related to the metabolism of carbohydrates, lipids, proteins, amino acids, nucleic acids, and nucleotides were significantly up-regulated; these included genes encoding for the mitochondrial hypoxia responsive protein, the mitochondrial co-chaperone GrpE, the glycoside hydrolase, the trypsin-like serine protease, the metalloprotease 1, the acetate transporter, the SGT1 and CS proteins, the formyltetrahydrofolate deformylase, the nucleoside triphosphate hydrolases, and uracil phosphoribosyltransferase [58]. On the basis of these findings, the strain degeneration mechanism in *C. militaris* is associated with genes involved in energy metabolism, toxin biosynthesis, DNA methylation, and chromosome remodeling [58].

DNA methylation refers to the transfer of methyl groups to a specific base by DNA methyltransferase, where S-adenosylmethionine acts as the methyl donor [65]. DNA methylation plays a key role in eukaryotic gene silencing and expression, phylogeny, cell differentiation, and other processes in eucaryotic organisms [66]. As regards fungi in particular, DNA methylation levels are relatively low, but of importance in degeneration phenomena observed after repeated subculturing, as is the case with *C. militaris*. Xin et al. [27] used a comparative bisulfite sequencing (BS-Seq) approach between a normal wild-type *C. militaris* strain and a degenerated one (incapable of producing fruit bodies) to establish that DNA the methylation level in the wild-type *C. militaris* was 0.48%; this value is lower than this of *Neurospora crassa* (1.5%) [67] but higher than that of *Magnaporthe grisea* (0.22%) [68]. Moreover, the methylation level of the degenerated type was higher than that of the wild-type (0.56% vs. 0.48%), while the degeneration of *C. militaris* cultures was associated with modifications in the pyruvate and glycerophospholipid metabolic, ubiquitin-mediated proteolysis, and N-glycan biosynthetic pathways [27].

## 3. Substrates, Nutrient Requirements and Treatments Related with the Cultivation of *C. militaris*

Despite the huge medicinal and economic value of *C. militaris* [2,69,70] there are relatively few studies addressing cultivation performance under various conditions. In addition, most of them focused on the low-scale laboratory production of *C. militaris* in order to examine factors affecting cordycepin biosynthesis but did not focus on large-scale industrial production, where parameters such as cost-effectiveness, substrate efficiency, cultivation time, and scalability should be also taken into consideration. 

Growth requirements under various cultivation conditions of *C. militaris* were recently assessed by using a genome-scale modeling (GEM) approach, which revealed interesting data about the core biochemical pathways and metabolic routes for this fungus [32]. *C. militaris* retains a conserved metabolism for efficiently producing ATP by mitochondrial oxidative phosphorylation in response to high-altitude environments. Moreover, it was found that *C. militaris* contains genes converting various hormones (e.g., serotonin, adrenaline, and dopamine) into amino acids; this is in accordance with the fact that in nature, *C. militaris* assimilates nitrogen and carbon from the larval corpus with high content in juvenile and neuronal hormones such as serotonin and dopamine (Table 2). Raethong et al. [32] concluded that *C. militaris*: (a) exhibit the highest growth rate on sucrose-based media, (b) could utilize a wide range of carbon and nitrogen sources, e.g., chitin, glucosamine, and gamma-aminobutyrate (GABA) under CN-limited conditions, (c) could not use cellulose as sole carbon source, (d) could utilize various nutrients and redirect them through the ammonia metabolism, while nitrogen content has a significant effect on the production of extracellular cordycepin, and (e) has an optimal C/N ratio for growth that is 12.7:1 (Table 2). It is worth noting that chitin and its derivatives are major components of insect exoskeletons, while rice grain is rich in GABA [32]. In line with the aforementioned findings, it was observed that chitosan (a chemical homologue of chitin) could significantly increase carotenoid content in a novel two-stage *C. militaris* cultivation process [71] (Table 2). In addition, low-nutrient growth media supplemented with insect cuticles increased the pathogenicity of *Beauveria bassiana*, *Cordyceps javanica*, and *Metarhizium robertsii* [33]. These effects are yet to be evaluated for *C. militaris*, but existing results have already proven a positive impact of insect-based compounds on its growth and productivity.

Considering fruit bodies production, a cost-effective substrate is mandatory in the development of relevant commercial applications. By-products of the agricultural and agro-industrial sectors are cheap, abundant, and suitable resources for the cultivation of edible and medicinal mushrooms [72,73,74,75]. However, relatively few studies have dealt with the production of *C. militaris* ascomata and bioactive compounds on such substrates. For example, the use of cottonseed shells or corn cobs at ratios of 8:1:1 (*w*/*w*/*w*) to wheat bran and rice resulted in higher fruit bodies yield compared with the use of conventional rice media, while corn cobs produced fruit bodies with the highest cordycepin content [12] (Table 2). In addition, it was observed that the addition of vegetable oils—such as those derived from soybean, peanut, rapeseed, olive, and palm, corn, and sunflower seeds—in static cultures of *C. militaris* significantly promoted mycelium growth; furthermore, peanut oil significantly increased cordycepin content as well [15] (Table 2). Starch-processing waste also proved to be suitable for mycelium growth of *C. militaris* under solid-state and submerged cultivation, but no information was provided regarding fruit bodies production in this substrate [11] (Table 2).

**Figure 1:**
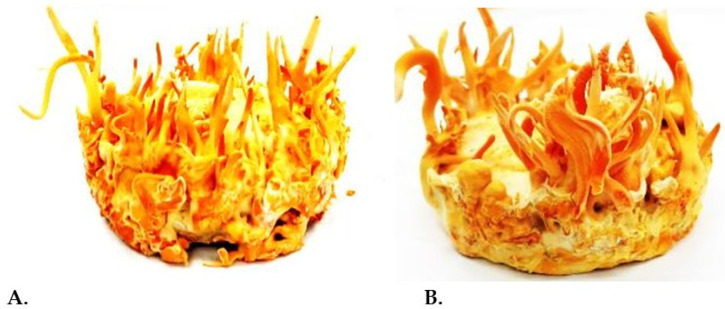
*C. militaris* strains producing ascomata following cultivation on rice-based substrates supplemented by insect tissues (Laboratory of General and Agricultural Microbiology, Agricultural University of Athens, Greece); (**A**) strain CmDK1, (**B**) strain CmDK2 [76].

Hence, it could be concluded that *C. militaris* benefits from substrates that emulate (or relate to) its insect hosts in nature. Therefore, a question arises as to whether plant-based media can be used alone for *C. militaris* cultivation or if insect-based supplements should be also added. Guo et al. [17] showed that there was no significant difference in yields of *C. militaris* following cultivation in wheat-based substrates and by injecting live *Bombyx mori* pupae. On the other hand, Wang et al. [34] observed significant variability in *C. militaris* mycelia produced on various liquid media and in fruit bodies cultivated on *B. mori* or rice-based substrates. The same authors detected the highest cordycepin content (13.43 mg g^−1^) in *C. militaris* fruit bodies produced after injection of fifth instar silkworm larvae 33 days post-infection. Furthermore, the outcome of ongoing studies revealed the beneficial effect of using rice-based substrates supplemented with insect tissues in the production of *C. militaris* ascomata (Figure 1) [76].

Other factors that influence *C. militaris* productivity include the use of low concentrations of fluoride (0.01 mM), which promoted fungal growth, increased superoxide dismutase-like activity, and enhanced biomass content in bioactive substances, including carotenoids [35] (Table 2). Moreover, extracts from *C. militaris* fruit bodies produced with the addition of fluoride exhibited a stronger anti-proliferation effect on U2OS cancer cells [35] (Table 2). 

Higher yields and biological efficiencies together with shorter fruit body maturation periods were observed in two degenerated *C. militaris* strains when their mycelia growing on the substrates surface were mechanically scratched by a metal scraper [36]. This scratching technique was used at various mycelium growth stages: (a) when mycelia entirely colonized the substrates eight days post inoculation in the dark, (b) when mycelia turned light yellow three days after the onset of illumination, and (c) when mycelia turned yellow and became twisted six days after the onset of illumination. Liu et al. [36] showed that scratching *C. militaris*-degenerated mycelia at these three vegetative stages and before stromata differentiation succeeded in shortening the period needed for growth of *C. militaris* fruit bodies by at least five days (Table 2). In addition, it was suggested that fruit bodies production by *C. militaris*-degenerated strains may be related to the *Rhf1* gene and the active oxygen-scavenging genes [36].

## 4. Genetics, Genomics, and Genetic Engineering of *C. militaris*

Genetic improvement of *C. militaris* is of high importance in order to create new strains with high commercial potential. As stated before, a breeder has to overcome several obstacles when attempting to improve *C. militaris* cultivation aspects, e.g., cultures degeneration, optimization of biomass and cordycepin production, reduction in time needed for fruit body maturation, determination of nutritional and environmental requirements, and assessment of fruit body composition. So far, *C. militaris* can be selectively improved by using traditional breeding techniques, and these are mainly restricted to reversing degenerative phenotypes to ascomata-producing strains. Fungal sexual reproductive systems are conservatively controlled by mating-type (MAT) loci [37,60,61]. Alternative sequences occupying the same chromosomal location at the MAT loci vary and have thereby been called idiomorphs or mini sex chromosomes [37,61,77,78]. It was suggested that the MAT genes play both convergent and divergent roles in mediating stroma development and fertility in *C. militaris* compared with the functions of MAT orthologs in other fungi [37]. Determination and comparison of cordycepin and adenosine production in fertile and sterile fruit bodies suggested that the fungal sexual process utilizes a significant amount of energy, thereby reducing the quality of the mushroom [37]. The mating types of *C. militaris* are controlled by a pair of alleles: *MAT1–1* (*MAT1–1–1*, *MAT1–1–2*) and *MAT1–2* (*MAT1–2–1*) [58,62,63]. Parent strains containing both *MAT1–1* and *MAT1–2* are considered heterokaryons, while parent strains containing only *MAT1–1* or *MAT1–2* are considered homokaryons [58,63]. Li et al. [64] by using Random Amplified Polymorphic DNA (RAPD) Polymerase Chain Reaction (PCR) analyses found that all strains incapable of developing normal fruit bodies were homokaryons, while all strains with normally developed fruit bodies were heterokaryons. Moreover, genotyping of degenerated *C. militaris* strains revealed that these strains contained a completely deleted *MAT1–2–1* region, which resulted in *CmMAT1–2–1* knockdown, base substitutions in the *MAT1–1–1* and *MAT1–2–1* regions, and *CmMAT1–1–1* and *CmMAT1–1–2* reduced expression [58]. Mating-based strain improvement has been achieved by crossing hyphae from spores of *MAT1–1* and *MAT1–2* idiomorphs, resulting in strains with high cordycepin content [79]. Moreover cross-mating with different mating types (rather than self-mating or crossing with the same mating types) decreases heterothallic limitation and increases the efficiency of industrial-scale *C. militaris* production [80]. 

Functional genomic studies may shed more light on factors affecting the development of *C. militaris* ascomata. Polyethylene glycol (PEG)-mediated transformation of *C. militaris* mononuclear protoplasts has been performed in order to knock-down a terpenoid synthase (*Tns*) gene using a glufosinate ammonium selection marker [48,49]. Using the same knockdown technique, it was revealed that the flavohemoprotein-like *Cmfhp* gene of *C. militaris* is involved in fruit body and conidia production, while it also affects nitric oxide (NO) and carotenoid contents [50]. Moreover, *Agrobacterium tumefaciens*-mediated transformation of *C. militaris* has been used as a tool for insertional mutagenesis [81] and complementation of *ΔCmfhp* mutants with the wild-type *Cmfhp* gene [50]. *Agrobacterium*-mediated transformation of *C. militaris* has also been used as a technique for deleting *MAT* genes in single mating-type isolates of this fungus [37]. Lu et al. [37] showed that *MAT1–1* and *MAT1–2–1* null mutants were sterile and lost the ability to produce stromata in outcrosses with the opposite mating-type partner, while *MAT1–1–1* produced barren stromata in outcrosses. Additionally, *MAT1–1–2*-generated fruit bodies were morphologically similar to that of the parent strain but with sterile perithecia [37]. The homothallic-like transformants *MAT1–2*:*MAT1–1–1* (haploidic *MAT1–2* isolate transformed with the *MAT1–1–1* gene) produced sterile stromata, whereas the *MAT1–1*:*MAT1–2–1* (haploidic *MAT1–1* isolate transformed with the *MAT1–2–1* gene) mutant lost the ability to produce ascomata [37]. The findings relating to the fully fertile gene-complementation mutants suggested that the genomic location is not essential for the *MAT* genes to fulfill their functions in *C. militaris* [37]. 

**Figure 2:**
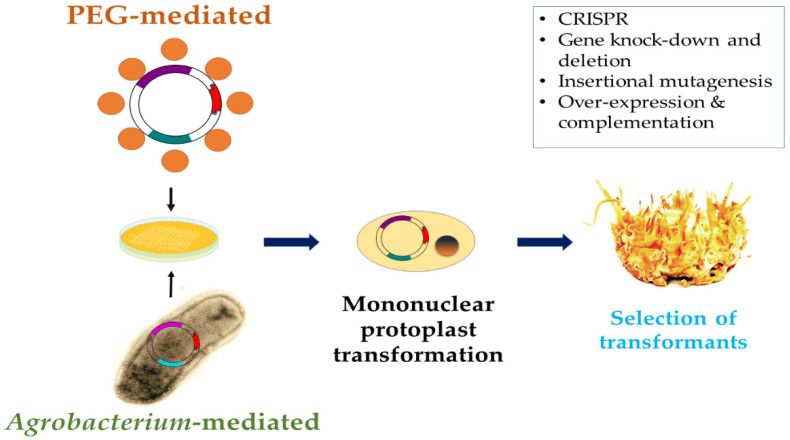
Schematic overview of genetic engineering approaches commonly used in *C. militaris*.

The clustered regularly interspaced short palindromic repeats (CRISPR) system is an innovative and efficient genome-editing tool that allows gene knockout, insertion, and replacement in almost all eukaryotic systems. Efficient CRISPR in *C. militaris* was achieved by creating a Cas9-stably transformed strain via *Agrobacterium*-mediated transformation and then by delivering of a presynthesized sgRNA targeting the *ura3* gene by PEG-mediated protoplast transformation [51]. In the same study it was reported that this method was superior to co-transformation of a single vector expressing a sgRNA-cmcas9 cassette, providing encouraging results for future interventions (genetic engineering) in the cordycepin biosynthesis pathway. A schematic overview of genetic engineering approaches used in *C. militaris* is provided in Figure 2.

*C. militaris* produces water-soluble carotenoid-containing pigments that are responsible for the characteristic yellow or orange color of the fruit bodies formed [82,83]. Although *C. militaris* pigments are secondary metabolites whose production is induced by light, not all light-induced genes are implicated in carotenoid biosynthesis. In a comparative transcriptome analysis approach using *C. militaris* mycelia exposed to light or darkness, Lou et al. [84] found a total of 1722 differentially expressed DEGs between these two conditions; therefore, only two genes, i.e., CCM_06728 (CAO-2) and CCM_09155 (YLO-1), could be associated with the biosynthesis of carotenoids. Unexpectedly, the expression levels of these genes were not significantly different between mycelia exposed to light and darkness [84]. The terpenoid synthase gene *Cmtns* seems to be involved in carotenoid biosynthesis of this fungus. Moreover, *ΔCmtns C. militaris* strains [48] showed a decrease in carotenoid content which was reversed by an *Agrobacterium*-mediated *Cmtns* complementation approach [84]; the reduced size of fruit bodies produced suggests that *Cmtns* might be a multifunctional gene.

*C. militaris* represents a valuable resource for isolating new natural compounds as products of its biosynthetic activity. The cyclodepsipeptide beauverolides were initially isolated and characterized from the entomopathogenic fungus *Beauveria tenella* in 1975 [85]; among them, beauveriolide I showed moderate insecticidal activities [86], while beauveriolide I and III showed specific inhibition of lipid droplet formation in mouse macrophages [87,88]. Moreover, beauveriolides inhibited acyl-CoA:cholesterol acyltransferases (ACATs) selectively, leading to a block in cholesteryl esters biosynthesis. Moreover, they led to a decrease in cholesterol concentration with no visible cytotoxic activities, making them candidate compounds for the production of antiatherosclerotic agents [89]. By using a genome mining approach on beauveriolides from *C. militaris* CM01, Wang et al. [90] identified one four-gene cluster (named cm3) related to cyclodepsipeptide biosynthesis in this fungus. After heterologous expression of the entire cm3 cluster into *Aspergillus nidulans* through the use of a protoplast transformation approach, beauveriolides I and III were detected at a concentration of 13 and 18 mg/L, respectively [90]. In the same study, the presence of both beauveriolides was evidenced in commercially produced fruit bodies of *C. militaris*.

## 5. Molecular, Nutritional, and Environmental Aspects of Cordycepin Biosynthesis and Ascomata Formation

As already mentioned, *C. militaris* produces a large number of bioactive metabolites, including ergothioneine, ergosterol, adenosine, polysaccharides, and cordycepin [14,91,92,93,94]. Among them, cordycepin exhibits a large range of health beneficial effects, such as broad-spectrum antibiotic activity and the ability to inhibit cell proliferation and to induce cell apoptosis, as well as having antioxidant, anticancer, and anti-inflammatory properties [95,96,97,98,99]. It is currently considered to be one of the most promising fungal compounds with respect to its pharmacological and therapeutic potential; therefore, the study of aspects related to cordycepin biosynthesis and ascomata production is essential to the commercial exploitation of this species.

The basic metabolic route for cordycepin biosynthesis has been recently explored using a combination of in silico analyses and functional genomics approaches [100]. Four genes for cordycepin synthetase were identified in *C. militaris* genome and were designated as *cns1*–*cns4*; these genes contain different conserved domains, such as the oxidoreductase/dehydrogenase domain in *Cns1*, the HDc family of metal-dependent phosphohydrolase domain in *Cns2*, and the N-terminal nucleoside/nucleotide kinase (NK) and C-terminal HisG domains in *Cns3*, while Cns4 is a putative ATP-binding cassette type of transporter. Using *Agrobacterium*-mediated single and joint gene deletion mutants of the aforementioned genes, Xia et al. [100] demonstrated that *Cns1* and *Cns2* are required for cordycepin biosynthesis, while *Cns3* is implicated in the biosynthesis of an additional “safeguard” molecule, i.e., pentostatin. In addition, the same authors reported that cordycepin biosynthesis (starting from adenosine and proceeding through the stepwise reactions of phosphorylation, dephosphorylation, and reduction) is catalyzed by the *Cns1*/*Cns2* complex in parallel with the biosynthesis of pentostatin. This dual production is similar to the bacterial “protector-protégé strategy” of purine metabolism in which the safeguarded cordycepin can be deaminated to 30-deoxyinosine once the former reaches a self-toxic level in fungal cells [100]. 

Sensing light is considered a signal for morphogenesis and metabolite production in fungi [82]. In the heterothallic filamentous fungus *Neurospora crassa*, the white collar (WC) complex consisting of the WC-1 and WC-2 proteins is the sensor for blue light [101]. WC-1 is a transcription factor implicated in all known blue light responses, including mycelial carotenogenesis, perithecial beak phototropism, circadian rhythms of conidiation, sexual development, and circadian clock resetting [82,102,103,104,105,106,107]. WC-1 contains a zinc finger DNA-binding domain, glutamine-rich putative transcription activation and protein–protein interaction domains, a nuclear localization signal, and a chromophore-binding domain [82,101]. WC-1 and WC-2 interact through the protein–protein interaction domains to form the functional white collar complex that binds to the promoters of light-regulated genes to rapidly activate transcription in response to light [82,104]. In *C. militaris*, *Cmwc-1* deletion resulted in disordered fruit body development and a decrease in conidial production, while it also led to a significant reduction in pigmentation as well as in cordycepin production; in addition, it affected spore formation and secondary metabolite production [82]. Moreover, in *Cmwc-1*-deletion mutants, 166 common genes were differentially expressed in response to light compared with the wild-type *Cmwc-1. C. militaris* was also found to contain homologs of the *CmWC-2*, *VVD*, *PHY*, and *CRY* (*CRY-1*, *CRY-2*, and *CPD photolyase*) genes, which confirms the importance of light perception in fruit body development [82]. Additionally, disruption of *Cmcry-DASH* resulted in completely different phenotypes than those of *ΔCmwc-1*, while *Cmcry-DASH* expression was strongly induced by light in a *CmWC-1*-dependent manner [108]. 

Albinism has also been implicated in the productivity of *C. militaris*. In a spontaneous albino mutation, with no color formation in either mycelia or ascomata, it was reported that albino mutants presented reduced conidial production and deformed fruit bodies of whitish color [109]. Comparative transcriptome analysis under light stress response showed that many more genes were expressed in the albino strain to reduce light impairment and that the significantly overexpressed pathways in the albino mutant were mainly involved in replication and repair mechanisms [109]. In addition, the expression levels of some secondary metabolite backbone genes were found to be differentially expressed by over 2-fold in the albino compared with the normal strains, e.g., the *Zn2Cys6* transcription factor and post-modification enzymes.

As previously mentioned with light stress, heat stress also influences cordycepin biosynthesis in *C. militaris*. It was suggested that during the late maturation stage of ascomata, heat and light stresses lead to a significant increase in cordycepin biosynthesis without affecting biological efficiency and that heat stress significantly promotes carotenoid production [110]. Moreover, it was observed that the optimal growth temperature for *C. militaris* is 20 °C on agar medium, while growth at 25 °C is compromised.

miRNA-like RNAs (milRNAs) have been discovered in various species of fungi with very little information with respect to their functions. RNA-dependent RNA polymerase (RDRP), argonaute (AGO), and Dicer are differentially expressed at different developmental stages of *C. militaris*, supporting the idea that the miRNAs pathways are present in this species [111]. High-throughput sequencing revealed the expression of 38 novel milRNAs; 19 are exclusively expressed in the sexual developmental stage, while the other 19 are expressed in both asexual and sexual developmental stages [111]. Two of them—the milR4 and milR16—were knocked out and over-expressed in *C. militaris*, and only the wild type and the overexpressed milR16 produced normal primordia and mature fruit bodies. Interestingly, yellow mycelia fully colonized pupae inoculated with the knocked miR-4 at 21 days post-injection, but this mutant was unable to form normal primordia.

A great variability in cordycepin content in ascomata and mycelia of *C. militaris* was assessed when pertinent literature was examined. Despite intense efforts to improve cordycepin production by *C. militaris* by modifying cultivation conditions and/or supplementing substrates with various nutrients, no widely accepted methodology has yet been established that could lead to consistently high cordycepin levels in the biomass obtained. Consequently, large fluctuations are commonly observed in cordycepin values measured either in mycelia generated from liquid fermentation (i.e., from 30 mg L^−1^ up to 8570 mg L^−1^) or in cultivated ascomata (i.e., from 0.6 mg g^−1^ up to 77.4 mg g^−1^) (Table 3). Such variability is attributed to various factors, one of the most important being the strain used. As it was previously presented, the genetic makeup of *C. militaris* seems to have a tremendous impact on both the ability to form ascomata and—most importantly—on the cordycepin biosynthetic capability of each individual strain [37,79,109] (Table 3.). Moreover, the analytical methodology adopted and the cordycepin extraction protocols employed to quantify cordycepin also confer highly diverse values that are difficult to interpret and associate with the fungus and the cultivation conditions used [38,39,112,113,114] (Table 3). Last, substrate composition could also exert a significant effect on cordycepin production, as evidenced by the relevant cases reported in Table 3 [40,41,42].

## 6. Conclusions

The ethnopharmacological importance of *C. militaris* has been widely analyzed in the past by many researchers [2,10,13,70,122,123,124] since this fungus is considered to be a valuable source of metabolites that act directly on various human metabolic pathways. For example, the methanolic extract of *C. militaris* presented antioxidant, antibacterial, antifungal, and antiproliferative properties in different human tumor cell lines [122], while cordycepin, the major bioactive compound of *C. militaris*, presented potent anti-inflammatory, anticancer, anti-metastatic, and immune-modulatory activities [123,124,125,126,127]. Although *C. militaris* can be easily cultivated in rice-based media, particular requirements related to the physiology and genetic makeup of this fungus impose serious obstacles in commercial applications. Hence, strain degeneration can completely hinder fruit body production at a commercial-scale, while improper cultivation techniques can downgrade metabolite production. Because of the entomopathogenic nature of *C. militaris*, the use of insect-based substrates in large-scale cultivation projects must be taken into consideration. During the past few years, insect mass production systems have become a contemporary trend in organic waste treatment and biotransformation [128], while the insect-based pet feed industry is growing exponentially. Considering the increasing availability of such by-products, the cultivation practices of *C. militaris* could exploit this type of waste material in the frame of the circular economy concept.

## Figures and Tables

**Table 1:** Factors that affect culture degeneration in *C. militaris*, their effect, and potential treatment.

Factors	Effects on Culture Degeneration: P: Promotes; H: Hinders	Treatment	References
**K^+^, Ca^2+^, Zn^2+^**	H	Addition in culture	[25]
**Mn^2+^, Mg^2+^**	P	Avoid usage	[25]
**Preservation**	H	Preservation at 4 °C	[28]
**Cellular accumulation of reactive oxygen species (ROS)**	P		[22]
**Subculturing**	P	Avoid subculturing after the third generation Rejuvenation by mating single ascospore isolates and producing new heterokaryons	[58]
**Culture temperature-medium**	P	Degenerated strains could be rejuvenated by using an insect host	[59]
**Homokaryosis**	P	Rejuvenation every 6 months Appropriate light regime during preservation	[21,24,26,29]
**DNA methylation**	P		[27,30]
**Genetic mutations**	P		[27,31]

**Table 2:** Basic requirements and cultivation practices affecting ascomata production or mycelium growth in *C. militaris*.

Substrates, Nutrients, and Treatments in the Cultivation of *C. militaris*	Cultivation-Related Practices	Effects on Ascomata Production or Mycelium Growth	References
Serotonin, adrenaline, and dopamine catabolism to amino acids	Addition of insect-based supplements in culture	Induces amino acids production	[32]
Chitin, glucosamine, and GABA catabolism in low C/N availability	Addition of insect-based supplements Addition of rice Addition of chitosan	Highest growth rate and improved cordycepin production	[32,33,34]
Sucrose utilization	Addition (instead of glucose)	Highest growth rate	[32]
C/N ratio	12.7:1	Optimal	[32]
Cottonseed shells, corn cobs particles at ratios of 8:1:1 (*w*/*w*/*w*) with rice and wheat	-	Increased fruit body yield and improved cordycepin levels	[12]
Vegetable oils	Addition in liquid culture	Cell growth and cordycepin production enhancement	[15]
Fluoride	Addition of 0.01 mM	Growth promotion and bioactive substance enhancement; stronger anti-proliferation effects of ascomata extracts on U2OS cancer cells	[35]
Aerial mycelia scratching	Scratching *C. militaris* degenerated mycelia before stromata differentiation	Shortening growth periods of *C. militaris* fruit bodies by at least 5 days	[36]

**Table 3:** Cordycepin production by *C. militaris* strains as measured in mycelia (M), fruit bodies (F), and liquid media (extracellular, L) under various cultivation conditions/treatments.

Strain Code (or N/A When No Strain Code Appears)	Cultivation Conditions	Type of Biomass and/or Substrate Measured	Cordycepin Concentration	References
Ν/A	Effects of various light wavelengths (darkness, red, pink blue, and daylight)	M	1.15–1.98 mg g^−1^	[115]
NBRC 9787	Addition of 1 g L^−1^ adenine plus glycine or l-glutamine in batch operation Addition of 1 g L^−1^ adenine plus glycine or l-glutamine repeated batch operation	M	19.7–33.7 mg L^−1^ 62.4–98.2 mg L^−1^	[43]
NBRC 9787 G81-3	Various strains and autoclaving techniques	M	76–237 mg g^−1^	[44]
Ν/A	Optimized carbon and nitrogen sources, i.e., 42.0 g L^−1^ glucose and 15.8 g L^−1^ peptone	M	Up to 345.4 mg L^−1^	[42]
KYL05	pH 6, 25 °C, 150 rpm, culture period of 6 days, carbon source: casein hydrolysate at 2%	M	Up to 445 mg L^−1^	[41]
Ν/A	pH 6, 25 °C, 110 rpm, culture period of 15–20 days, addition of 1 g L^−1^ FeSO_4_ at day 0	M	Up to 596.6 mg L^−1^	[40]
CICC 14014	Addition of 30 g L^−1^ peanut oil in standard medium	M	Up to 5290 mg L^−1^	[15]
Ν/A	Effects of sodium selenite (0–18 ppm)	F	0–0.6 mg g^−1^	[116]
CGMCC2459	Effect of various mineral salts: K_2_HPO_4_, KH_2_PO_4_, Ca(NO_3_)_2_, CaCl_2_, KCl, MgSO_4_7H2O, FeSO_4_	F	0.95–5.72 mg g^−1^	[117]
CGMCC33.16322	Wheat standard substrate and pupal (*B. mori*) injection	F	~1 mg g^−1^ ~1.2 mg g^−1^	[17]
CGMCC2459	Effect of different nitrogen sources (wheat bran, soybean oil meal, beef extract, peptone, yeast extract, silkworm pupa, NH_4_NO_3_)	F	1.78–10.90 mg g^−1^	[117]
CGMCC2459	20 g of brown rice, millet, sorghum, corn, wheat, and glutinous rice as fruiting medium supplemented with 32 mL of nutrient solution	F	2.42–5.62 mg g^−1^	[117]
CGMCC2459	Effect of various growth factors (vitamins B1, B9, α-naphthyl acetic acid, 2,4-dichlorophenoxyacetic acid, indole-3-butytric acid	F	2.92–6.21 mg g^−1^	[117]
CGMCC3.16321	Generation of 498 (CGMCC 5.2190) sibling normal strain, generation of 505 (CGMCC 5.2191) albino strain by spontaneous mutation	F	3.09 mg g^−1^ for the normal sibling strain 6.70 mg g^−1^ for the albino strain	[109]
CGMCC2459	Effect of various carbon sources (glucose, sucrose, amidulin, lactose, maltose, mannose)	F	3.77–6.50 mg g^−1^	[117]
CGMCC2459	Effect of initial pH (5.0–8.0)	F	4.39–7.40 mg g^−1^	[117]
CGMCC 3.16321	Heat stress treatment at 25 °C, light at 1700 lx	F	Up to 5.56 mg g^−1^	[110]
KSP8	Single spore mating of SPNU 1006xKACC44455	F	Up to 6.63 mg g^−1^	[118]
N/A	Addition of tea leaves or *Andraca theae* droppings in basal media	F	8.35–12.85 mg g^−1^	[119]
Cm09	Generation of ΔMAT1-1-2; injection of 10^7^ ΔMAT1-1-2xΜAΤ1-2 spores/mL into the Chinese Tussah silkworm pupae	F	Up to 16.77 mg g^−1^	[37]
NO. 20130508	Corn cob particles/wheat bran/rice bran (8:1:1) + 20 g L^−1^ glucose, and 5 g L^−1^ peptone Cottonseed shells/wheat bran/rice (8:1:1) + 20 g L^−1^ glucose, and 5 g L^−1^ peptone; 20 g rice+ 20 g L^−1^ glucose, and 5 g L^−1^ peptone	F	26.9 mg g^−1^ 23.4 mg g^−1^ 34.5 mg g^−1^	[12]
Ν/A	20 g rice and 20 mL potato dextrose medium + selenate or selenite or selenomethionine at a concentration of 40 μg g^−1^ in rice	F	43.3 mg g^−1^ 69.3 mg g^−1^ 77.4 mg g^−1^	[45]
CM10	Basal medium: 20 g L^−1^ peptone, 24.7 g L^−1^ sucrose, 1.11 g L^−1^ K_2_HPO_4_⋅3H_2_O, 0.90 g L^−1^ MgSO_4_⋅7H_2_O, 0.01 g L^−1^ vitamin B1 plus 8 g L^−1^ L-alanine, and overexpression of CCM_02568 and CCM_01481 transcription factors	L	30.04–99.83 mg L^−1^	[23]
Ν/A	Liquid fermentation in basal media containing silkworm pupae powder, wheat, or silkworm pupae powder, plus wheat by applying different extraction methodologies (heat, frequency, solvents, and resins)	L	Up to 39.40 mg L^−1^	[112]
CCRC 32219	Multifactorial analysis (four factors): pH 4 to 7, various nitrogen sources, varying yeast extract content, shake vs. static conditions	L	44.4–1375.6 mg L^−1^	[46]
TBRC6039	Rational design of synthetic media	L	65.7–377 mg L^−1^	[32]
GACP08Y5 GACP08Y1 GACP0746	20 g L^−1^ sucrose, 20 g L^−1^ peptone, 1 g L^−1^ KH_2_PO_4_, and 0.5 g L^−1^ MgSO_4_·7H_2_O in static liquid bioreactors of different culture volumes	L	271–4376 mg L^−1^	[118]
NBRC 9787	Addition of 1 g L^−1^ adenine plus glycine or l-glutamine in batch operation Addition of 1 g L^−1^ adenine plus glycine or l-glutamine repeated batch operation	L	542.4–2500 mg L^−1^ 3400–14,100 mg L^−1^	[43]
CGMCC2459	Optimized medium (20 g L^−1^ peptone, 24.7 g L^−1^ sucrose, 1.11 g L^−1^ K_2_HPO_4_·3H_2_O, 0.90 g L^−1^ MgSO_4_·7H_2_O, 10 mg L^−1^ vitamin B1, 5.45 g L^−1^ hypoxanthine, and 12.23 g L^−1^ L-alanine)	L	Up to 2008 mg L^−1^	[118]
NBRC 9787 G81-3	Various strains and autoclaving conditions	L	2400–10,900 mg L^−1^	[44]
BCRC34380	Effect of porcine liver extracts (0.5 g L^−1^, 1 g L^−1^, 5 g L^−1^, 7.5 g L^−1^, and 10 g L^−1^)	L	Up to 2452 mg L^−1^	[12]
BCRC34380	Effect of blue light irradiation (0, 8, 16, and 24 h d^−1^)	L	Up to 3483 mg L^−1^	[12]
NBRC 103752	72.5 g L^−1^ yeast extract, 62.6 g L^−1^ glucose (pH 5.6), and Vogel’s medium at 1:10 concentration	L	Up to ~5000 mg L^−1^	[120]
CM14014	60 g L^−1^ glucose, 0.7 g L^−1^ KH_2_PO_4_, 0.7 g L^−1^ MgSO_4_ 7H_2_O, 9.00 g L^−1^ yeast extract, and 17.10 g L^−1^ tryptone at 27.1 °C; seed age, 3 days; inoculum size, 10%	L	Up to ~7350 mg L^−1^	[47]
NBRC 9787	Surface liquid culture and mutagenesis by ion beam irradiation	L	Up to 8570 mg L^−1^	[38]
CM016	Solid state fermentation on rice-based medium containing 20 g L^−1^ sucrose, 10 g L^−1^ peptone, 0.1 g L^−1^ MgSO_4_ 7H_2_O, and 0.1 g L^−1^ KH_2_PO_4_ using a four-factor, five-leveled central composite against glucose, peptone, adenine, histidine	F	1.92–20.86 mg g^−1^	[121]

## Data Availability

Not applicable.
